# Unclassifiable Renal Cell Carcinoma With an Estimated 100% Sarcomatoid Component: A Case Report

**DOI:** 10.7759/cureus.74984

**Published:** 2024-12-02

**Authors:** Chaimae Bekhakh, Anass Haloui, Nada Akouh, Karich Nassira, Amal Bennani

**Affiliations:** 1 Department of Anatomopathology, Mohammed VI University Hospital/Faculty of Medicine, Mohammed 1st University, Oujda, MAR

**Keywords:** carcinoma, kidney, rare entity, sarcomatoid, unclassifiable

## Abstract

Sarcomatoid renal cell carcinoma (RCC) is an aggressive tumour with a poor prognosis. It is not a distinct histological entity, as it can be found in any subtype of renal cell carcinoma. The majority of cases will present with advanced or metastatic disease requiring systemic treatment. However, pathologists are sometimes confronted with renal cell tumours that do not fit any definition, so these tumours must be considered unclassified renal cell carcinomas. The criteria for this classification are varied and include tumours with more than one histological subtype, tumours with unrecognized epithelial histological subtypes, mucin-producing tumours, and tumours with an exclusively sarcomatoid or rhabdoid morphology. The latter subgroup of unclassified renal cell carcinomas is rare in renal pathology. Consequently, the presence and quantity of the sarcomatoid component must be reflected in pathology reports. We report the case of a 76-year-old male patient presenting with intermittent left lumbar pain for two months, in a deteriorating general condition. Histological and immunohistochemical examinations revealed an unclassifiable renal cell carcinoma with an estimated 100% sarcomatoid component.

## Introduction

Renal cell carcinomas (RCCs) account for numerous variations and subtypes of malignant tumours that originate from the epithelial cells of kidney tissue. The latest WHO classification of primary renal epithelial neoplasms has, for the most part, well defined these subtypes morphologically and immunohistochemically, dividing them into 14 histological subtypes and 4 provisional entities. However, pathologists on occasion are faced with renal cell tumours that fail to properly fit into any one definition; these tumours would then have to be regarded as unclassified renal cell carcinomas. The criteria for this classification are varied and include tumours that show more than one histological subtype, tumours with unrecognised epithelial histological subtypes, mucin-producing tumours, and tumours that show an exclusive sarcomatoid or rhabdoid morphology. This final subgroup of unclassified renal cell carcinomas is rare in renal pathology [1].

Owing to their rarity and their peculiar morphological manifestation, these tumours can pose a diagnostic issue for unfamiliar pathologists. We report here the case of a male patient with a highly aggressive renal cell tumour with exclusively sarcomatoid morphology.

## Case presentation

**Figure 1:**
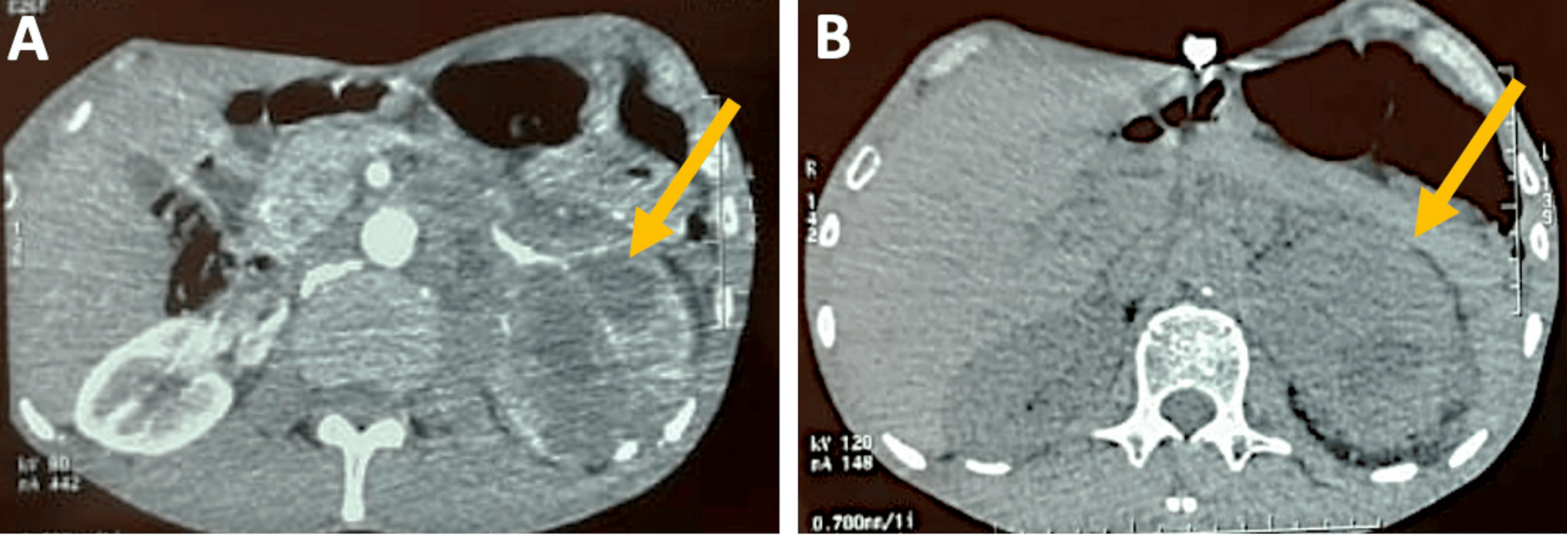
Irregularly contoured and heterogeneous mass with solid and necrotic areas located in the left kidney's upper pole.

We report the case of a 76-year-old male smoker with no significant medical history. The patient has been experiencing intermittent left lumbago for the past two months, leading to a decline in their overall health. A CT scan was performed, showing an enlarged left kidney with an irregularly contoured and heterogeneous upper pole mass measuring 54 mm × 42 mm, containing areas of necrosis and tissue enhancement post-contrast injection (Figure 1).

**Figure 2:**
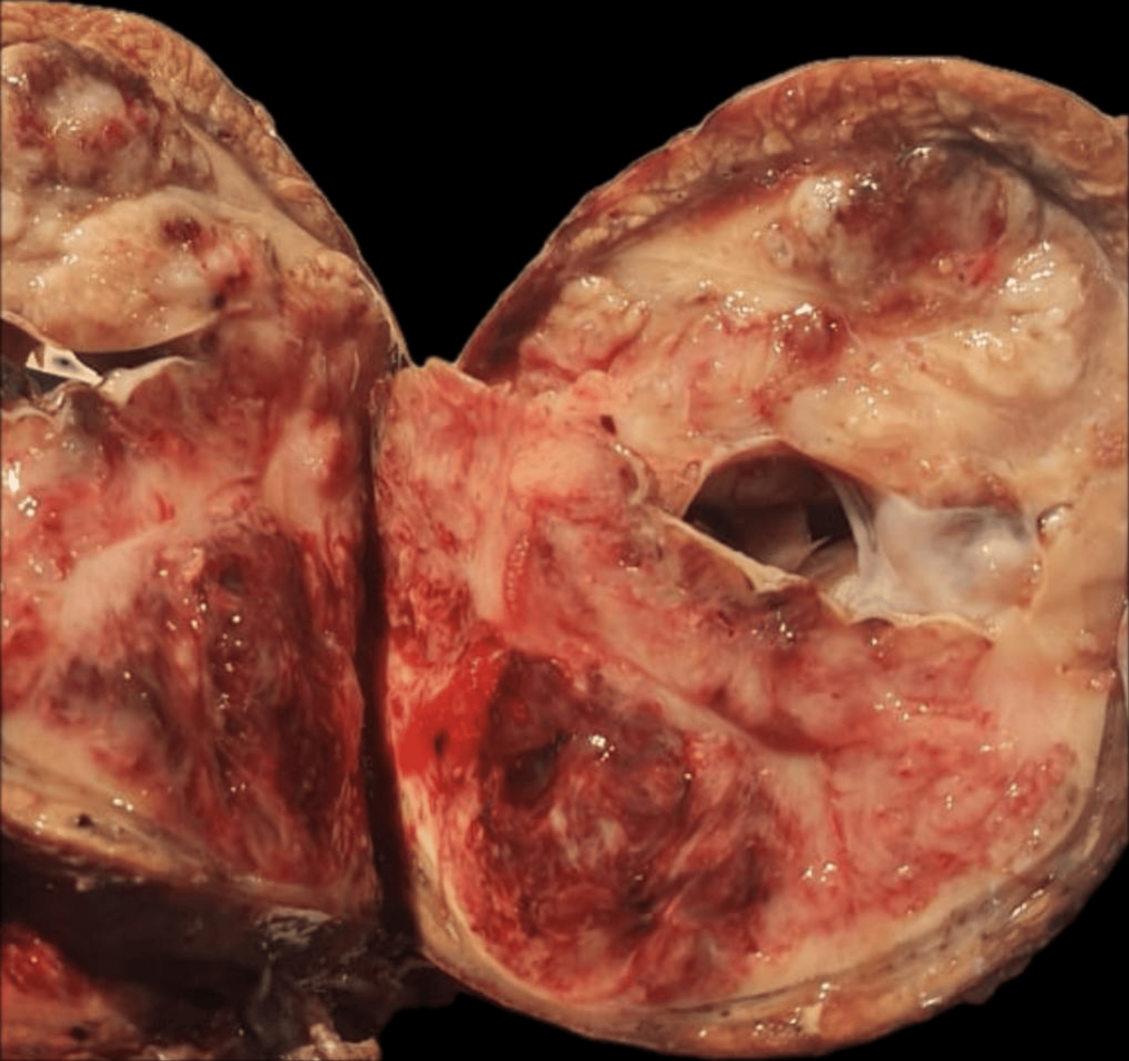
Macroscopic appearance of the left kidney mass.

The patient underwent an enlarged total left nephrectomy. A gross examination of the specimen revealed a solid, well-circumscribed beige lesion occupying the entirety of the kidney, measuring 12 cm × 10 cm × 8 cm, with associated haemorrhagic and myxoid zones​​ (Figure 2).

**Figure 3:**
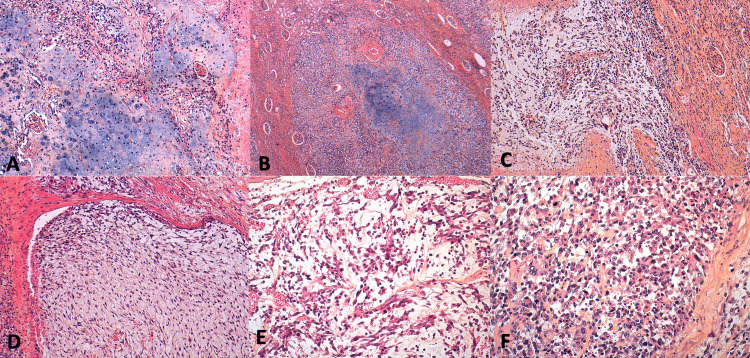
The different sarcomatous components. (A & B) Cartilaginous component; (C & D) fusocellular component; (E) vascular component; (F) rhabdoid component.

Microscopically, the various samples showed an intra-renal tumoral proliferation separated from the renal parenchyma by a fibrous capsule with a mostly diffuse and focally vaguely nodular architecture, with alternating hypo- and hypercellular zones. Tumour cells were medium-sized, ovoid, or spindle-shaped. They showed moderate cytonuclear atypia with nucleolated, irregularly contoured hyperchromatic nuclei and abundant clear or eosinophilic cytoplasm. Tumour necrosis is present, and the tumour stroma was fibro-inflammatory and myxoid. Various sarcomatous components were present, notably cartilaginous, rhabdoid, osseous, and vascular (Figure 3).

**Figure 4:**
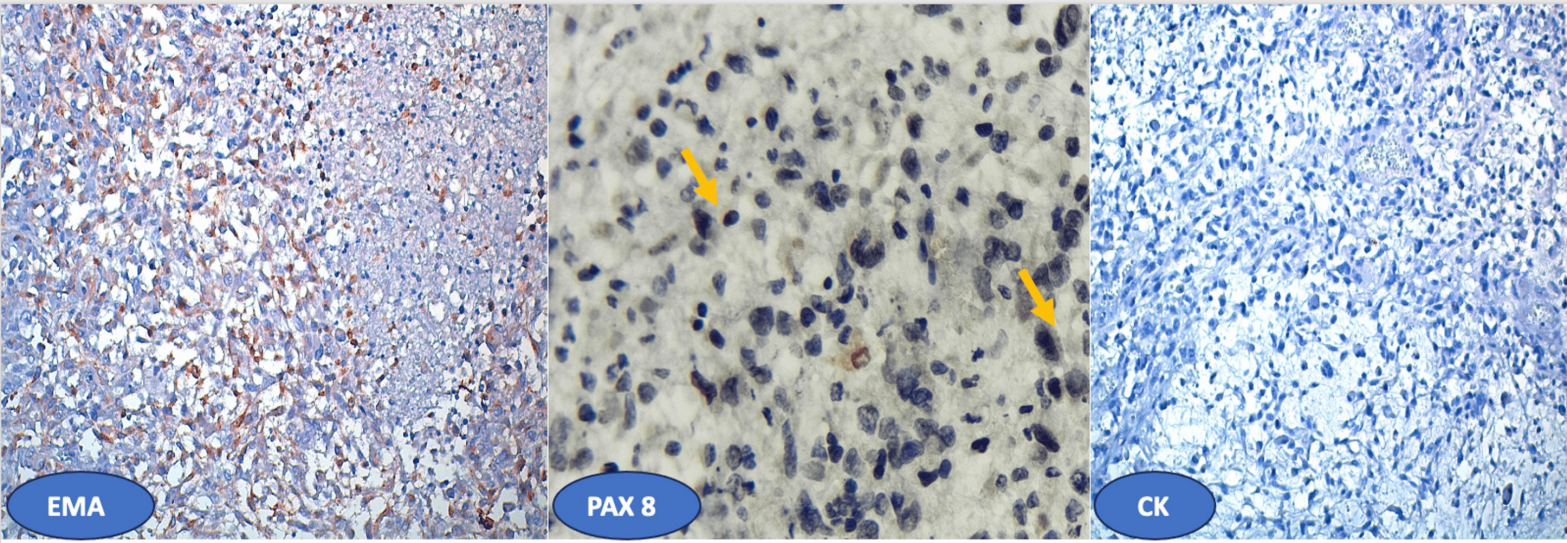
EMA and PAX 8: heterogeneous, focal positive labelling of tumour cells/CK: no marking.

An immunohistochemical study showed heterogeneous, focal expression of PAX 8 and epithelial membrane antigen (EMA) by tumour cells. However, they do not express pancytokeratin (Figure 4).

Overall, the diagnosis of purely sarcomatoid unclassified renal cell carcinoma was retained. Taking into consideration the age of the patient, the deterioration of his general condition, and the presence of distant metastases (pulmonary nodules), the therapeutic decision was to put the patient on palliative care (chemotherapy). At the time of writing, the patient has unfortunately succumbed to the adverse effects of chemotherapy and subsequently passed away.

## Discussion

Unclassified renal cell carcinoma is a heterogeneous group of neoplasia that fails to fall under the definition of any one of the fourteen histological subtypes of renal cell carcinoma or the four provisional histological subtypes previously defined by the latest WHO classification of primary renal epithelial neoplasms. Failure to fit the requirements of diagnosis for these subtypes is not entirely uncommon, reported to have an incidence that can reach up to 5.7% of all renal cell carcinomas. Neoplasms may require this designation if more than one histological subtype is present, if mucin production is present, or if an unrecognized epithelial histological subtype is present. Lastly, tumours that show exclusive sarcomatoid or rhabdoid morphology without any recognizable epithelial component also require this designation. In Perrino and colleagues' study, they grouped unclassified renal cell carcinomas further into three subgroups based on their histological aspects [1]: (i) group 1 - oncocytoma/chromophobe RCC-like; (ii) group 2 - clear cell RCC-like; (iii) group 3 - others (i.e., papillary RCC-like/collecting duct-like/pure sarcomatoid).

Sarcomatoid dedifferentiation is not a rare find in RCCs, with incidence rates that vary depending on the primary epithelial subtype and range from 5.2% to 8% in ccRCC, 1.9% to 5.4% in papillary RCC, 2% to 9% in chromophobe RCC, and 25% to 29% in collecting duct RCC [2]. The exact physiopathology of this phenomenon has not yet been fully explored and understood. However, several studies support the theory of a common cell of origin for the sarcomatoid cells and the epithelial cell component owing to the fact that these two components share many similarities in their genetic profile. Further proof is the immunohistochemical staining pattern of these sarcomatoid cells, which retain to a certain degree the expression of both epithelial markers (pancytokeratin and EMA) and renal cell markers (PAX8, CD10, and CAIX). Sarcomatoid cells also show a loss of expression of e-cadherin; meanwhile, they gain expression of N-cadherin and vimentin along with an increase in the levels of the Snail transcription factor, all of which supports the claim that these cells may be engaging in epithelial-mesenchymal transition (EMT) [3]. Sarcomatoid dedifferentiation confers a more aggressive behaviour to tumours and is in itself enough of a criterion to classify clear cell and papillary cell RCCs as ISUP grade 4 regardless of the percentage of tumour tissue dedifferentiating [3]. This percentage can also vary between patients, with patients rarely presenting with 100% dedifferentiated tumours without any identifiable primary epithelial subtype, rendering the identification of the epithelial nature of these tumours highly challenging. After extensive sampling and elimination of a potential primary sarcoma of the kidney, metastasis, or the direct involvement of retroperitoneal sarcoma (leiomyosarcoma and liposarcoma being the chief contenders in these suspicions), these rare and purely sarcomatoid tumours require the designation as unclassified renal cell carcinomas [1]. In this case report, we encountered such a tumour that exhibits a 100% sarcomatoid dedifferentiation, multiple areas showed differing types of dedifferentiation such as vascular, fibrous, and osseous, which was taken as a supportive argument towards a dedifferentiating carcinoma rather than a sarcoma exhibiting all these different paths of dedifferentiation, such a wide variety of dedifferentiation is a rarity, as most sarcomatoid renal cell carcinomas exhibit features of malignant fibrous histiocytoma or fibrosarcoma [4]. The nephrectomy piece was extensively sampled with consistent findings throughout and a distinct lack of any potential epithelial origin of the sarcomatoid proliferation. In addition to that, the immunohistochemical studies of the proliferation showed a profile that is consistent with an epithelial origin with positive, albeit discrete, epithelial membrane antibody staining. Positive PAX8 staining was considered proof of the renal origin of the proliferation. In addition to that, although the proliferative cells also stained for multiple antibodies that provided proof of different sarcoma-like differentiation processes occurring, such as CD31, CD34, SMA, etc., these stains were focal at most, with no geographical overlap being reserved to the locations where the proliferation showed the appropriate dedifferentiation for the proper stain.

The proliferation showed an extensive invasion of the renal structures and the surrounding fat tissue, which is compatible with the aggressive nature of these tumours, which warrants their high ISUP grade [3-4].

In terms of treatment, for localized cases, surgery remains the gold standard, but participation in an adjuvant therapy trial should be considered because of the high risk of recurrence.

In the case of metastases, chemotherapy combined with antiangiogenic therapy could play an important role, although survival is generally short. At the same time, better molecular and genetic characterization of sarcomatoid renal cell carcinoma will enable us to better understand this entity and develop specific therapies [5]. In our case, the patient was a candidate for palliative care and unfortunately died.

## Conclusions

Unclassified renal cell carcinomas are a heterogeneous group of proliferations that fail to fit into the established definitions of the variants of renal cell carcinoma accepted by the World Health Organisation. Carcinomas that show an exclusive sarcomatoid morphology and lack an epithelial component are rare entities that require being grouped into unclassified renal cell carcinomas, however, such a term poses certain diagnostic pitfalls in this particular case as it requires both proof of the lack of a potential epithelial low-grade origin through extensive sampling and the elimination of a primitive or metastatic sarcoma. These tumours retain to a certain extent the expression of epithelial-origin antibodies (pancytokeratin and/or EMA) and renal-cell-origin antibodies (PAX8, CD10, CAIX) though they may be focal and weak. The rarity of these tumours coupled with their aggressive nature and the diagnostic difficulties pathologists may encounter when facing them makes raising awareness of such an event highly important.
